# Per capita interactions and stress tolerance drive stress-induced changes in biodiversity effects on ecosystem functions

**DOI:** 10.1038/ncomms12486

**Published:** 2016-08-18

**Authors:** Jan M. Baert, Colin R. Janssen, Koen Sabbe, Frederik De Laender

**Affiliations:** 1 Laboratory of Environmental Toxicology and Applied Ecology, Ghent University, Jozef Plateaustraat 22, 9000 Ghent, Belgium; 2 Laboratory of Protistology and Aquatic Ecology, Ghent University, Krijgslaan 281-S8, 9000 Ghent, Belgium; 3 Research Unit of Environmental and Evolutionary Biology, University of Namur, Rue de Bruxelles 61, 5000 Namur, Belgium

## Abstract

Environmental stress changes the relationship between biodiversity and ecosystem functions, but the underlying mechanisms are poorly understood. Because species interactions shape biodiversity–ecosystem functioning relationships, changes in per capita interactions under stress (as predicted by the stress gradient hypothesis) can be an important driver of stress-induced changes in these relationships. To test this hypothesis, we measure productivity in microalgae communities along a diversity and herbicide gradient. On the basis of additive partitioning and a mechanistic community model, we demonstrate that changes in per capita interactions do not explain effects of herbicide stress on the biodiversity–productivity relationship. Instead, assuming that the per capita interactions remain unaffected by stress, causing species densities to only change through differences in stress tolerance, suffices to predict the stress-induced changes in the biodiversity–productivity relationship and community composition. We discuss how our findings set the stage for developing theory on how environmental stress changes biodiversity effects on ecosystem functions.

## Abstract (web summary)

Biodiversity often increases ecosystem functions, but stressors could disrupt that relationship. Using a microalgae model, Baert *et al.* show how stress-induced changes in the biodiversity–ecosystem function relationships depend on species interactions and stress tolerance.

## Graphical abstract

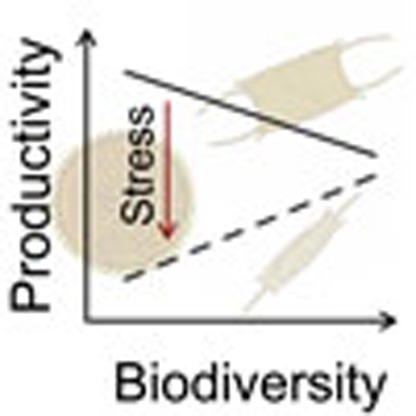

Since the 1990s, hundreds of empirical studies established that biodiversity affects ecosystem functions^1^,^2^,^3^,^4^,^5^,^6^. Today, there is mounting empirical evidence that stress caused by changes in environmental conditions alters the biodiversity–ecosystem function relationship. However, observations have been inconsistent among studies. While the majority of studies reported a decreased effect of biodiversity on ecosystem functions with increasing stress^7^,^8^,^9^,^10^,^11^,^12^,^13^,^14^,^15^,^16^, others reported no change^17^,^18^ or even an increase^19^. The mechanisms underlying these stress-induced changes in biodiversity effects—and possibly explaining the observed differences among studies—remain virtually unexplored. This lack of mechanistic understanding hampers our ability to predict the value of biodiversity in the many ecosystems that are currently challenged by environmental stress^20^,^21^.

Biodiversity effects on ecosystem functions are driven by species interactions. When inter- and intraspecific interactions differ in strength, biodiversity affects ecosystem functions as species will function differently in the presence of other species compared with their monocultures^22^,^23^,^24^. Ecological theory distinguishes two classes of biodiversity effects. First, interspecific interactions can change species contributions to ecosystem functions because of competitive replacement. This dominance effect alters ecosystem functions because of the increased functional contribution of superior competitors^23^. Second, interspecific interactions can also change species functional contributions without resulting in competitive replacement. Such effects are referred to as complementarity effects as they are mainly attributed to niche complementarity or facilitative interactions between species^23^.

Species interactions are not only important determinants of biodiversity effects. They also regulate how stress will alter species' contributions to ecosystem functions^25^,^26^,^27^. Stress has a direct effect on species densities through effects on species fitness (reproduction and/or survival)^28^,^29^. Species interactions determine the extent by which these direct effects on species densities will affect other species^26^,^27^.

Species interactions thus take up a central position in both theory on biodiversity–ecosystem function relationships and stress ecology^22^,^23^,^25^,^26^,^27^. Understanding whether and to what extent stress affects species interactions is therefore crucial for the development of theory on stress-induced changes in biodiversity effects. Existing theories make conflicting predictions on the effect of environmental changes on the per capita strength of species interactions^30^. The stress gradient hypothesis proposes a shift in the per capita interaction strengths from competitive to facilitative interactions when environmental conditions become more stressful (that is, reduce fitness) to prevent fast competitive exclusion^31^. Coexistence theory, in contrast, does not predict any direct effect of stress on per capita species interactions. Hence, stress is assumed to alter the effect of species interactions by causing species-specific effects on fitness^32^,^33^. Both theories, by consequence, make different predictions on how stress can modulate biodiversity effects.

If the per capita strength of species interactions is unaffected by stress, as assumed by coexistence theory, changes in biodiversity effects only result from the direct effects on species fitness and the same per capita interactions occurring in unstressed conditions. Hence, stress should principally change biodiversity effects on ecosystem functions through changes in dominance because of the replacement of sensitive by stress-tolerant species, as the latter by definition grow better when stressed. If, however, per capita interactions become more positive under stress, as predicted by the stress gradient hypothesis, also complementarity is expected to increase with stress.

Understanding how stress changes the effects of biodiversity on ecosystem functions is essential for ecosystem management but remains as yet virtually unexplored^7^,^9^,^16^. Here we examine how stress caused by the herbicide Atrazine affects dominance and complementarity effects on productivity in marine diatom microcosms. We therefore measure community composition and biovolume production in marine diatom communities along a diversity and herbicide (Atrazine) gradient in microcosms. We test what changes in biodiversity effects drive stress effects on the shape of the biodiversity–ecosystem function relationship, and whether stress effects on the per capita strength of species interactions contribute to these changes. Two different approaches are used: (1) a partitioning method to quantify dominance and complementarity effects (2) a mechanistic community model. Both approaches strongly support the absence of stress effects on per capita species interaction strengths. Instead, we find that interspecific variability stress tolerance and the strength of per capita species interactions in unstressed conditions can explain how stress alters biodiversity effects on ecosystem functions. Finally, we discuss how our results are the first step towards a mechanistic theory explaining how environmental stress can change biodiversity effects on ecosystem functions in a variety of study systems.

## Results

### Microcosm experiment

**Figure 1:**
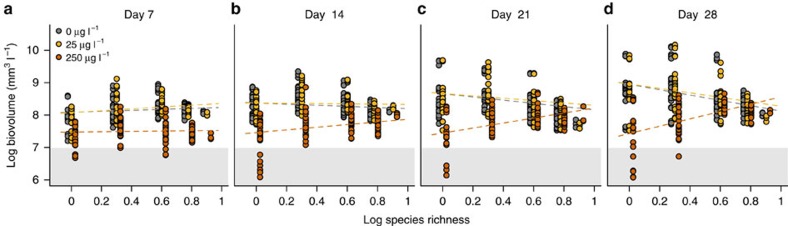
Stress-induced changes in the biodiversity–ecosystem functioning relationship. Log_10_ biovolume for 39 diatom communities spanning five levels of species richness at (**a**) day 7, (**b**) 14, (**c**) 21 and (**d**) 28 of the experiment for control (grey), low stress (yellow) and high stress (orange) conditions. Regression lines represent the predicted biodiversity–productivity relationship by the linear mixed effects model (Table 1). The grey area corresponds to negative net biovolume production.

**Figure 2:**
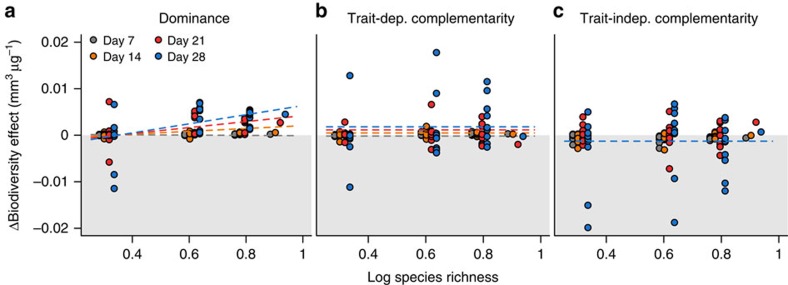
Stress-induced changes in biodiversity effects. Changes in (**a**) dominance, (**b**) trait-dependent and (**c**) trait-independent complementarity effect at day 8 (grey), 14 (orange), 21 (red) and 28 (blue) of the experiment. Regression lines correspond to the predicted stress-induced changes biodiversity effects by the linear mixed effects models using species richness, atrazine concentration and day as predictor variables (Table 2, model 1). Note that regression lines overlap for the trait-independent complementarity effect.

Atrazine application changed the shape of the biodiversity–ecosystem function relationship (Fig. 1). Biodiversity decreased biovolume production in control and low stress (25 μg l^−1^ atrazine) conditions, but increased biovolume production at elevated stress (250 μg l^−1^ atrazine) conditions. Throughout the whole experiment there was no significant difference between the no stress and low stress treatment (Table 1). Atrazine had larger effects on biovolume production at lower richness (Table 1). The biodiversity–ecosystem function relationship thus shifted from negative to positive under stress because of reduced stress effects on productivity in more species-rich communities (Fig. 1). This effect of stress on the slope of the biodiversity–ecosystem function relationship was entirely driven by corresponding changes in dominance (Fig. 2, Supplementary Fig. 1). Only atrazine-induced changes in the dominance effect increased with species richness, and increased over time (Table 2). Atrazine effects on complementarity effects, in contrast, occurred independent of species richness for both trait-dependent and trait-independent complementarity effects (Fig. 2, Table 2).

### Community model

**Figure 3:**
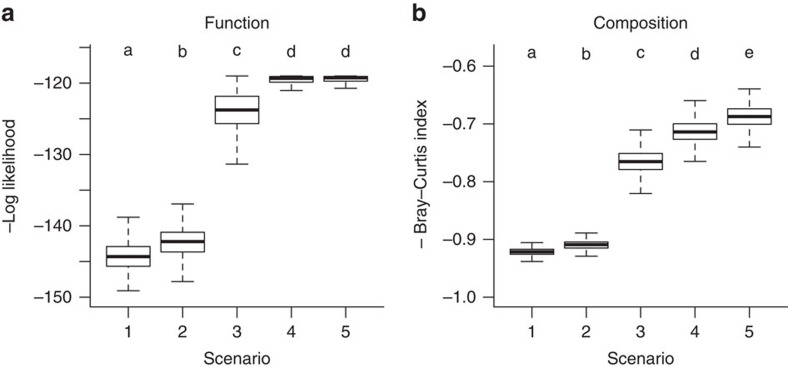
Community model predictions under different scenarios of stress effects. Boxplots of the negative log-likelihood of the change in community productivity (**a**) and average negative Bray–Curtis dissimilarity index (**b**) for five scenarios of stress-induced effects in the per capita strength of species interactions. Scenario 1 is the baseline scenario without interspecific interaction (*A*_ij_=0). Scenario 2 corresponds to equal inter- and intraspecific interaction strength (*A*_ii_=*A*_ij_). Scenario 3 corresponds to a constant ratio of inter- to intraspecific competition (*A*_ii,0_/*A*_ij0_=*A*_ii250_/*A*_ij250_). Scenario 4 corresponds to a constant per capita strength of interspecific interactions (*A*_ij0_=*A*_ij250_). Scenario 5 did not impose any limitations to changes in per capita interaction strength. Higher values indicate a better model fit and scenarios that do not share a common letter are significantly different (Bonferroni-corrected Wilcoxon signed-rank test: *P*<0.05). Boxplot whiskers correspond to maximal 1.5 times the interquartile range.

In our microcosm study, the biodiversity–ecosystem function relationship changed because ecosystem functions were better buffered in more diverse systems (Fig. 1). This result was driven by increased dominance by stress-tolerant species under stress (Fig. 2, Supplementary Fig. 1). We used a community model (Fig. 3) to test to what extent atrazine effects on the per capita species interactions are needed to reproduce these two main patterns observed in our data: diversity-dependent buffering of atrazine effects and dominance shifts. We compared an extensive set of model simulations, representing five scenarios making different assumptions on stress effects on per capita interactions, to these two patterns. This analysis indicated that there is no conclusive support for stress effects on per capita species interactions. Allowing for effects on per capita interactions did not significantly improve the model's fit to the observed stress effects on ecosystem functions (Wilcoxon signed-rank test: *W*_999_=533537, *P*=0.09, Fig. 3a). The predicted effects of atrazine on composition were highly similar between scenarios that assumed fixed (scenario 3 and 4) and changing per capita interaction strengths (scenario 5). Allowing for atrazine effects on interaction strengths improved the model fit by only 3% (Wilcoxon signed-rank test: *W*_999_=808299, *P*<0.001, Fig. 3b). The direct effects of atrazine on species fitness by reducing growth (that is, as observed in monocultures) combined with the per capita species interactions for unstressed conditions (scenario 4) sufficed to predict the function and composition in stressed microcosms (Fig. 3, Supplementary Fig. 2).

### Stress-induced changes in biodiversity effects

**Figure 4:**
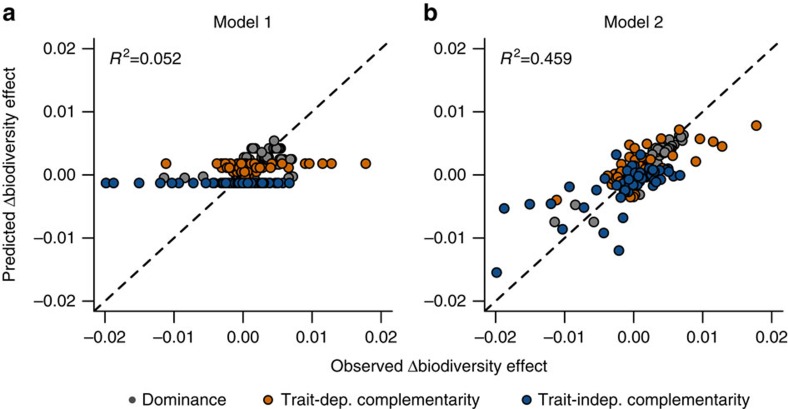
Predicted stress-induced changes in biodiversity effects. Predicted plotted against observed changes in the dominance (grey), trait-dependent complementarity (orange) and trait-independent complementarity effect (blue) for linear regression models (**a**) including diversity and day (Table 2, model 1) and (**b**) the weighted mean strength of per capita interactions and weighted mean species stress tolerance as predictor variables (Table 2, model 2).

Atrazine affected only the dominance effect in a way that depended on species richness (Fig. 2). Atrazine also affected both complementarity effects, but not in a way that depended on richness. In fact, much of the among-community variation in the changes of the complementarity effects was left unexplained (Fig. 2, Fig. 4a). We tested to what extent changes in biodiversity effects depended on direct stress effects on species growth (established in monoculture bioassays), the strength of per capita interaction in unstressed conditions (estimated under scenario 4) and stress effects on these interactions (estimated under scenario 5) could explain this variation. Estimated effects on per capita interaction strengths did not significantly explain the variation in any of the biodiversity effects (Supplementary Table 1). Instead, direct stress effects on species growth and the strength of per capita interactions in unstressed conditions explained 46% of the observed variation in the observed changes in biodiversity effects (Fig. 4b, Table 2).

## Discussion

Confirming other studies^7^,^9^,^13^,^14^,^15^,^16^, we found that envionmental stress changed the biodiversity–ecosystem function relationship (Fig. 1, Table 1). We demonstrated that stress effects on the per capita strength of interspecific interactions, if occurring at all, did not contribute in any ecologically meaningful way to such change. We base this conclusion on three lines of evidence. First, the change in the biodiversity–ecosystem function relationship was clearly not driven by stress effects on complementarity effects (Fig. 2). Second, direct stress effects on species fitness, that is, the growth reduction in monoculture bioassays, sufficed to predict the observed stress effects on ecosystem function and community composition with a mechanistic community model. This finding mechanistically demonstrates that stress effects on the biodiversity–ecosystem function relationship were mainly driven by direct effects on species growth (Fig. 3). Allowing for stress effects on the per capita interaction strength did not significantly improve the model's capacity to predict effects of stress on ecosystem functions (Fig. 3a). While allowing for such effects improved model predictions of community composition, this improvement (3%) was smaller than the variability among replicates (5%). Hence, this improvement merely reflects a different number of free parameters between scenarios and the extremely high power when sample sizes are very large (*n*=31,000). This improvement thus does not indicate an ecologically relevant improvement of model fit. Third, the direct effect of atrazine on species growth and the strength of species interactions estimated in unstressed conditions could explain the variability in the biodiversity effects among systems (Fig. 4).

The positive effect of stress on the slope of the biodiversity–ecosystem function relationship can be expected in many different communities and is no specific feature of our study system. Indeed, the insurance hypothesis^34^,^35^ postulates that diverse communities are more likely to contain species that can thrive under stress and buffer ecosystem functions by replacing sensitive species^34^,^36^. Therefore, functions that are merely the sum of individual species contributions should be affected less by stress in more diverse systems and the slope of the biodiversity–ecosystem function relationship should increase. This is exactly what we found: functional replacement and thus the dominance effect increased with diversity (Fig. 2 and Supplementary Fig. 1), and atrazine affected production less in more diverse communities (Fig. 1).

We show that stress can not only affect the slope of the biodiversity–ecosystem function relationship by changing dominance but also through shifts in complementarity (Fig. 4). Because the sign and size of these shifts depend on the interspecific per capita interaction strengths in unstressed conditions (Table 2), these shifts are most likely system-specific. Depending on the strength of these interactions in a study system, complementarity shifts can counteract, offset or add to the general effect stress has on dominance. Differences in interaction strengths among studies can thus potentially lead to different effects of stress on the biodiversity–ecosystem function relationships^13^,^19^.

In this study, we used planktonic microalgae, which generally experience strong interspecific competition because of limited spatial heterogeneity^37^. Algal community performance is therefore often determined by the dominant species, and frequently leads to negative dominance^38^,^39^,^40^, and even a negative biodiversity–ecosystem function relationship^41^. Such a negative relationship in unstressed conditions amplifies the positive effect of biodiversity on the buffering of ecosystem functions, shifting the relationship from negative to positive under stress. Studies with terrestrial systems, in contrast, often reported positive biodiversity–ecosystem function relationships that are driven by strong complementarity effects^9^,^12^,^14^,^22^. So, even though studies that quantified biodiversity effects reported an increased dominance effect through environmental changes, the overall slope decreased because the increasing dominance effect was outweighed by a decrease in complementarity effects^9^,^14^.

The findings presented in this study thus offer a first step towards a mechanistic understanding how environmental stress alters the biodiversity–ecosystem function relationship. Our results suggest that dominance effects can generally be expected to increase under stress by changes in fitness through interspecific differences in stress tolerance. However, if per capita interactions remain unaffected, stress does not necessarily increase complementarity effects, as expected based on the stress gradient hypothesis. Therefore it is unlikely that stress affects biodiversity–ecosystem function relationships and the underlying biodiversity effects in a general way as previously suggested^7^. Instead, stress effects can strongly depend on the species interactions, specific to the study system. System specific conservation efforts may therefore be required to preserve the services provided by the many ecosystems that currently suffer from environmental stress factors, including organic chemicals such as pesticides^21^,^42^.

## Methods

### Algal strains

Diatoms were isolated from a single phytoplankton sample collected near the Thorntonbank (Southern bight of the North Sea) during the spring bloom in March 2013. Single cells were isolated from the sample using a micropipette. Next, the cells were rinsed three times with growth medium and cultured as monoclonal stock cultures^43^. F/2 medium^44^ based on artificial seawater (salinity 33±1‰; Instant Ocean) and supplemented with 30 μg l^−1^ Si was used as growth medium. Stock cultures were maintained in an acclimatized room (20±1 °C) at a 12 h photoperiod and a 35±5 μmol photons m^−2^ s^−1^ light intensity (Lumilux 18W cool white Osram). New cultures were inoculated weekly to sustain exponential growth. The photoperiod was prolonged to 16 h two weeks before the start of the experiment.

### Microcosm experiment

We randomly selected eight strains belonging to different species (*Bacillaria sp., Coscinodiscus sp., Ditylum sp., Guinardia sp., Gyrosigma sp., Odontella sp.* and two strains of *Thalassiosira sp.*) differing in size, division rate and stress tolerance (Supplementary Table 2). Communities of five levels of species richness were represented at each of the three levels of atrazine (that is, a full-factorial design). To separate species-identity from diversity effects^45^, 10 different random assemblages were made at each richness level, except at levels 1 and 8 where only 8 and 1 assemblages were possible (Supplementary Table 3). Atrazine concentrations (0, 25 and 250 μg l^−1^) that represented a control, low stress and high stress treatment were selected from preliminary tests. Microcosms were established in three replicates at each concentration (351 microcosms in total).

Communities were inoculated in sterilized 100 ml glass Erlenmeyer flasks filled with 35 ml F/2 medium containing the required atrazine (Sigma-Aldrich) concentration, and fitted with cellulose plugs. Species were inoculated at an equal proportion of the total initial biovolume (10^7^ mm^3^ l^−1^). To minimize variability between replicates and assemblages, species were inoculated from single stock cultures. Microcosms were cultured for 4 weeks at 20± 1 °C and a 35±5 μmol photons m^−2^ s^−1^ 16 h photoperiod. Weekly, 80% of the growth medium was renewed to avoid nutrient limitation or stress reduction through the atrazine photolysis. It was verified that cell number in the removed growth medium did not exceeded 0.1% of the total biovolume. To determine species densities, 1 ml samples were taken, fixed with formaldehyde at a 6% final concentration, and stored at 4 °C in 24-well plates until analysis. Cell densities were determined using an inverse microscope and Whipple grid. Only living cells were counted. Mortality could easily be assessed on the basis of empty frustules (that is, the empty siliceous diatom cell walls which remain after the cells have died, see Supplementary Fig. 3). Mortality rates were very low, independent of the diversity treatment. In nearly all communities, dead cells accounted for less than 1% of the total cells. Species that were completely inhibited by atrazine, however, showed an increased relative proportion of dead cells in the high stress treatment because population dynamics were only driven by mortality (see Supplementary Table 2). Biovolumes were calculated on the basis of the average cell volume of each species, calculated from a sample of 50 cells^46^. To verify constant stress levels and the absence of nutrient limitation, we weekly determined nitrate, phosphate and silicate concentrations spectrophotometrically (Aquamate, Thermo Electron Corporation + Spectroquant test kits, Merck Millipore) and atrazine concentrations by GC-MS (Thermo Quest Finnigan Trace DSQ coupled to Thermo Quest Trace 2000 series).

### Calculation of biodiversity effects

Biodiversity effects were calculated using an additive tri-partite partitioning method^23^. This method is based on the comparison of the observed yield of a species in mixture to that expected under the null-hypothesis that inter- and intraspecific competition are equal. Under this null hypothesis, species performance is independent of diversity. Hence, species are expected to realise a proportion of their monoculture yield (that is, ‘observed relative yield', RY_O_) equal to their initial proportion in the mixture (that is, ‘expected relative yield', RY_E_). This species-specific expected yield allows to factor out potential confounding effects related to differences in species composition effects (for example, sampling effects)^47^. The partitioning splits the deviation of the total mixture yield from that expected under the null-hypothesis (Δ*Y*) in dominance, trait-dependent complementarity and trait-independent complementarity effects:


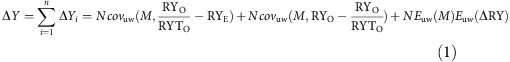


These three biodiversity effects reflect how the individual species yields (Δ*Y*_i_) deviate from the null hypothesis, and whether deviations depend on species functional abilities (that is, the monoculture yield *M*). The first term, the dominance effect, quantifies the extent by which species deviate from the null hypothesis by replacing other. This is measured by the unweighted covariance (that is, not accounting for the species' initial proportion in the mixture) between a species monoculture yield and the deviation of its realized fraction of the relative yield total, RYT_O_ (that is, as if the species where competing within a zero-sum game) from that expected under the null-hypothesis (that is, RY_E_). The second term, the trait-dependent complementarity effect, quantifies the extent by which species' deviations from null hypothesis that do not results competitive replacement (that is, deviates from a zero-sum game) correlate to the monoculture yield. The third term, the trait-independent complementarity effect, is the product of the average monoculture yield and the average species deviation from the null hypothesis, and quantifies to what extent species deviate on average from the null hypothesis, irrespective of their monoculture yield.

### Data analysis

Linear mixed effects models were used assess the effects of log_10_ diversity (LDiv), atrazine concentration (*C* with factor levels control, C0, low stress, LS, and high stress, HS) and time (Day) on the log_10_ biovolume, and of log_10_ diversity and time on stress-induced changes in biodiversity effects (i.e. dominance, trait-dependent complementarity and trait-independent complementarity effects) were evaluated using linear mixed effects models. Full models included all possible predictor interactions:


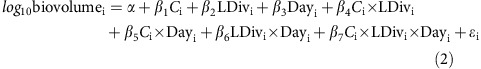



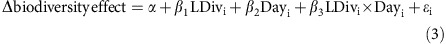


Models were optimized through a backward selection procedure. Interactions were only retained when main effects were significant or when removing them did no longer result in normal distributions of model residuals. Because of the temporal dependence of the data, full models were fitted with a continuous first-order autocorrelation structure. Community assemblage was included as a random effect to account for species identity effects. Models that incorporated community assemblage as a random effect (that is, a random incept model) were significantly better than those without (analysis of variance (ANOVA): *F*_14,13_=628, *P*<0.001). Temporal autocorrelation structures, in contrast, were only required for models predicting changes in biodiversity effects (ANOVA: *F*_7,6_=5.3, *P*<0.05). Validity of the optimal models was assessed on the basis of the normality of model residuals (Supplementary Figs 4–11).

Next, we tested to what extent stress-induced changes in biodiversity effects depended on direct stress effects on species growth, the strength of per capita interaction in unstressed conditions and stress effects on these interactions. These predictors were respectively quantified as the mean weighted atrazine effects on monoculture growth (*M*_250_/*M*_0_), the per capita interaction coefficients in unstressed conditions (*A*_0_) and the atrazine effects on the per capita interaction coefficients (*A*_250_−*A*_0_), which were estimated by the community model (see the next section). All the estimates were weighted for the relative species abundance in the control treatment. Initial full models included all pairwise interaction effects:


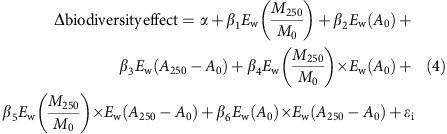


where *E*_w_ represents the weighted mean and *ɛ*_i_ the model residuals. Community composition was included as a random effect (ANOVA: *F*_9,8_=22.1, *P*<0.0001). Model residuals were not temporally correlated. Optimal models were obtained from a backward selection procedure and normality of model residuals was assessed (Supplementary Figs 12–14). Analyses were conducted in R 3.1.1. (ref. 48) using the lme4 package^49^. Only changes day 21 and 28 were included since strong biodiversity–ecosystem function relationships only developed after 14 days (Fig. 1). Estimates of species monoculture growth in unstressed (*M*_0_) and high-stress conditions (*M*_250_) and absolute interspecific competition coefficients (log *A*_i,j_) were obtained from the community model (see the next section). Model estimates under scenario 4 were used for the fixed strength of per capita interactions, whereas estimates under scenario 5 were used for the change in per capita interaction strength (see parameter estimation).

### Community model

A Lotka–Volterra model with a stress-dependent intrinsic growth rate and carrying capacity was used to simulate community dynamics:


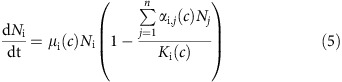


Where *N*_i_ (μm^3^ l^−1^) is the biovolume density, *μ*_i_ (d^−1^) is intrinsic growth rate and *K*_i_ (μm^3^ l^−1^) is the carrying capacity of species i, *α*_i,j_ (−) is the interaction coefficient between species i and j, *n* is the total number of species and *c* is the atrazine concentration (μg l^−1^). This equation can also be rewritten in terms of absolute competition coefficients *A*_i,j_ (*c*)=*α*_i,j_(*c*) *K*_i_(*c*)^−1^:


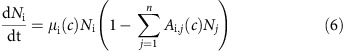


### Community model simulations and evaluation

Model parameters were optimized (see the next section) under the restrictions of five different scenarios to test for stress-induced changes in per capita interaction strength (Table 1). The first scenario is a baseline scenario without interspecific interactions (that is, *α*_i,j_(*c*)=0 or *A*_i,j_(*c*)=0). Species densities thus only depend on the stress-effect on their demographic rates in this scenario. In the second scenario, per capita inter- and intraspecific interaction strength are assumed to be equal (that is, *α*_i,i_(*c*)*=α*_i,j_(*c*)=1 or *A*_i,i_(*c*)=*A*_i,j_(*c*)). Hence, community dynamics still only result from interspecific variability of stress effects on growth. In the third scenario, the ratio between the strength of inter- and intraspecific interaction is constant (that is, *α*_i,i_(*c*)/*α*_i,j_(*c*)=constant or *A*_i,i_(*c*)/*A*_i,j_(*c*)=constant). The strength of per capita interactions, however, increases when stress decreases the species' maximum function *K*_i_(*c*). In the fourth scenario, absolute strength of the per capita interactions are assumed to be constant (that is, *A*_i,j_(*c*)=constant). In the fifth scenario, species interactions are allowed differ between stress levels without any assumptions.

In each scenario, the upper and lower limits of *μ*_i_(*c*) and *K*_i_(*c*) were constrained within 10% of the value estimated for the monocultures. When growth rates were lower than 0.1 d^−1^, the upper limit were set to 0.15 d^−1^ to avoid too stringent conditions when parameters values are underestimated from the monoculture data. Despite this correction, estimated values never exceeded the monoculture value by more than 30%. Relative interaction coefficients were limited between 0 and 200 based on the estimated values from diversity level 2. Because the control and 25 μg l^−1^ treatment were not significantly different (Fig. 1, Table 1), parameters were only estimated for the control and 250 μg l^−1^ atrazine treatment. Parameters were estimated 100 times for each scenario to estimate parameter uncertainty. Next, 1,000 Monte Carlo simulations were ran for each scenario sampling. For each run, parameters were randomly from a uniform distribution constrained by the 2.5 and 97.5 percentile of parameter estimates. The average species density of each community at the start of the experiment was used as initial densities for model simulations. Community densities were simulated for 28 days, analogous to the experiment. Scenarios were compared using the likelihood of the proportion functional lost (that is, 
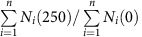
) and the average Bray–Curtis similarity between observed and predicted community compositions (that is, 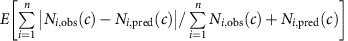) for each Monte Carlo run. The likelihood based on species densities evaluates how well the model predicts stress-induced changes in ecosystem function; the average Bray–Curtis similarity evaluates stress-induced changes in community composition. Comparisons were made by a signed-rank test with Bonferroni correction. All simulations were performed in R 3.1.1. (ref. 48) using the GenSA package^50^.

### Community model parameter estimation

Optimal parameter values were estimated using a simulated annealing optimization algorithm and the time and density weighted mean average percentage error (MAPE) as objective function. The MAPE was selected as objective function because biovolumes could differ by eight orders of magnitude between species in a community. Therefore an objective function that scaled model deviations was required to ensure a comparative goodness of fit for all species (that is, a good prediction of community composition). The MAPE was weighted for the relative species abundance to ensure a good prediction of the total community biovolume in (highly) uneven communities and was weighted for the sampling day to deal with the larger uncertainty on the low densities at day 7 and 14 biovolumes which could exceed the values expected from the per capita growth rate in some species. The final objective function *S* can thus be written as:


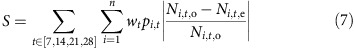



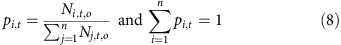



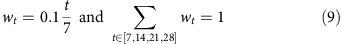


where *N*_*i,t,*o_ is the observed biovolume of species *i* at time *t*, *N*_*i,t,*o_ is the expected biovolume, *p*_*i,t*_ is the relative species abundance at time *t*, *w*_*t*_ is the weight of time *t*, and *n* is the number of species in the community.

To ensure an efficient exploration of the parameter space, parameter sets that resulted in species densities reaching infinity, extinction of more than one species ore the MAPE exceeding 100% we penalized by setting the objective function to:


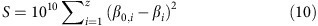


where 
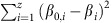
 is the Euclidean distance of the parameter set (*β*_*1*_*,… β*_*z*_) from the initial parameter values (*β*_*01*_*,… β*_*0z*_) of the optimization algorithm. This ensures that the algorithm returns to the initial parameters when it runs into a series of irrelevant solutions.

In addition, to avoid over fitting of the model by unrealistically high interaction coefficients, the mean value of the interaction effect of each species was assumed not to exceed 1,000 times the average species abundance. When the mean value exceeded this cut-off value, the excess was added to the objective function. This favours a reduction of species density either by a reduction in carrying capacity or by competition with abundant species rather than by competition with rare species.

### Code availability

The R code to generate the figures, simulations and analyses presented in this study are available online at https://github.com/JanBaert/Per-capita-interactions-and-stress-tolerance-drive-stress-induced-changes-in-biodiversity-effects.

### Data availability

All the data supporting the findings presented in this study are available within the article and its Supplementary Information files (Supplementary Data 1 and Supplementary Table 4).

## Additional information

**How to cite this article:** Baert, J.M. *et al.* Per capita interactions and stress tolerance drive stress-induced changes in biodiversity effects on ecosystem functions. *Nat. Commun.* 7:12486 doi: 10.1038/ncomms12486 (2016).

## Supplementary Material

Supplementary Information Supplementary Figures 1-14 and Supplementary Tables 1-4

Supplementary Data 1 Biovolume densities at day 7, 14, 21 and 28 of the experiment for 8 species of marine diatoms in 39 communities at three different atrazine concentrations

## Figures and Tables

**Table 1:** Biodiversity and stress effects on log_10_ biovolume.

	DF	*t* value	Estimate (s.e.)	*P* value
Intercept	1,355	77.8	7.799 (0.10)	<0.0001
LDiv	37	2.56	0.475 (0.19)	0.0144
Day	1,355	12.4	0.041 (0.003)	<0.0001
LS	1,355	−0.2	−0.016 (0.09)	0.8560
HS	1,355	−3.5	−0.307 (0.09)	0.0006
LDiv × Day	1,355	−7.6	−0.046 (0.006)	<0.0001
LDiv × LS	1,355	0.8	0.140 (0.17)	0.3968
LDiv × HS	1,355	−4.5	−0.785 (0.17)	<0.0001
LDiv × LS × Day	1,355	−0.9	−0.008 (0.008)	0.3672
LDiv × HS × Day	1,355	10.9	0.098 (0.008)	<0.0001

Mixed effects model estimating the effect of log_10_ diversity (LDiv), 25 μg l^−1^ (LS) and 250 μg l^−1^ (HS) atrazine stress on the log_10_ biovolume over the course of the experiment (Day). s.e. is the standard error of the estimated fixed effects.

**Table 2:** Stress-induced changes in biodiversity effects.

	Dominance effect				Trait-dependent complementarity effect				Trait-independent complementarity effect			
	DF	*t* value	Estimate (s.e.)	*P* value	DF	*t* value	Estimate (s.e.)	*P* value	DF	*t* value	Estimate (s.e.)	*P* value
**Model 1**												
Intercept	91	0.94	0.0014 (0.002)	0.35	92	−0.91	−0.0008 (0.0009)	0.36	93	−2.078	−0.00127 (0.0006)	0.04
LDiv	29	−1.47	−0.0037 (0.003)	0.15								
Day	91	−2.54	−0.0002 (0.0001)	0.013	92	2.07	0.0001 (0.0001)	0.04				
LDiv × Day	91	4.28	0.0005 (0.0001)	<0.0001								
												
**Model 2**												
Intercept	29	2.40	0.022 (0.009)	0.02	29	0.94	0.011 (0.01)	0.35	29	3.46	0.044 (0.01)	0.002
Tol	27	−2.41	−0.097 (0.04)	0.02	27	−3.02	−0.164 (0.05)	0.006	29	3.60	0.005 (0.001)	0.001
Inter	27	2.34	0.003 (0.001)	0.03	27	0.94	0.001 (0.001)	0.36				
Tol × Inter	27	−2.48	−0.012 (0.005)	0.02	27	−3.06	−0.020 (0.006)	0.005				

Model 1: Mixed effects model estimating the effect of log_10_ diversity (LDiv) on stress-induced changes in biodiversity effects over the course of the experiment (Day). Model 2: Mixed effects model estimating the effect of the weighted mean species stress tolerance (Tol) and per capita strength of interspecific interactions (Inter) on changes in biodiversity effects at day 21 and 28. Means were weighted for the relative species abundance. Tolerance was calculated as the ratio between the species monoculture yield at 250 μg l^−1^ atrazine and in control conditions. The strength of interspecific interactions was based on the parameter estimates of the community model (see the ‘Methods' section). s.e. is the standard error of the estimated fixed effects.
