# Supplementary material for: Per capita interactions and stress tolerance drive stress-induced changes in biodiversity effects on ecosystem functions
Source: Nat Commun. 2016 Aug 18;7:12486. doi: 10.1038/ncomms12486 (PMC4992148; doi:10.1038/ncomms12486)
Supplement: Supplementary Information — Supplementary Figures 1-14 and Supplementary Tables 1-4 [file ncomms12486-s1.pdf]

Supplementary Figures

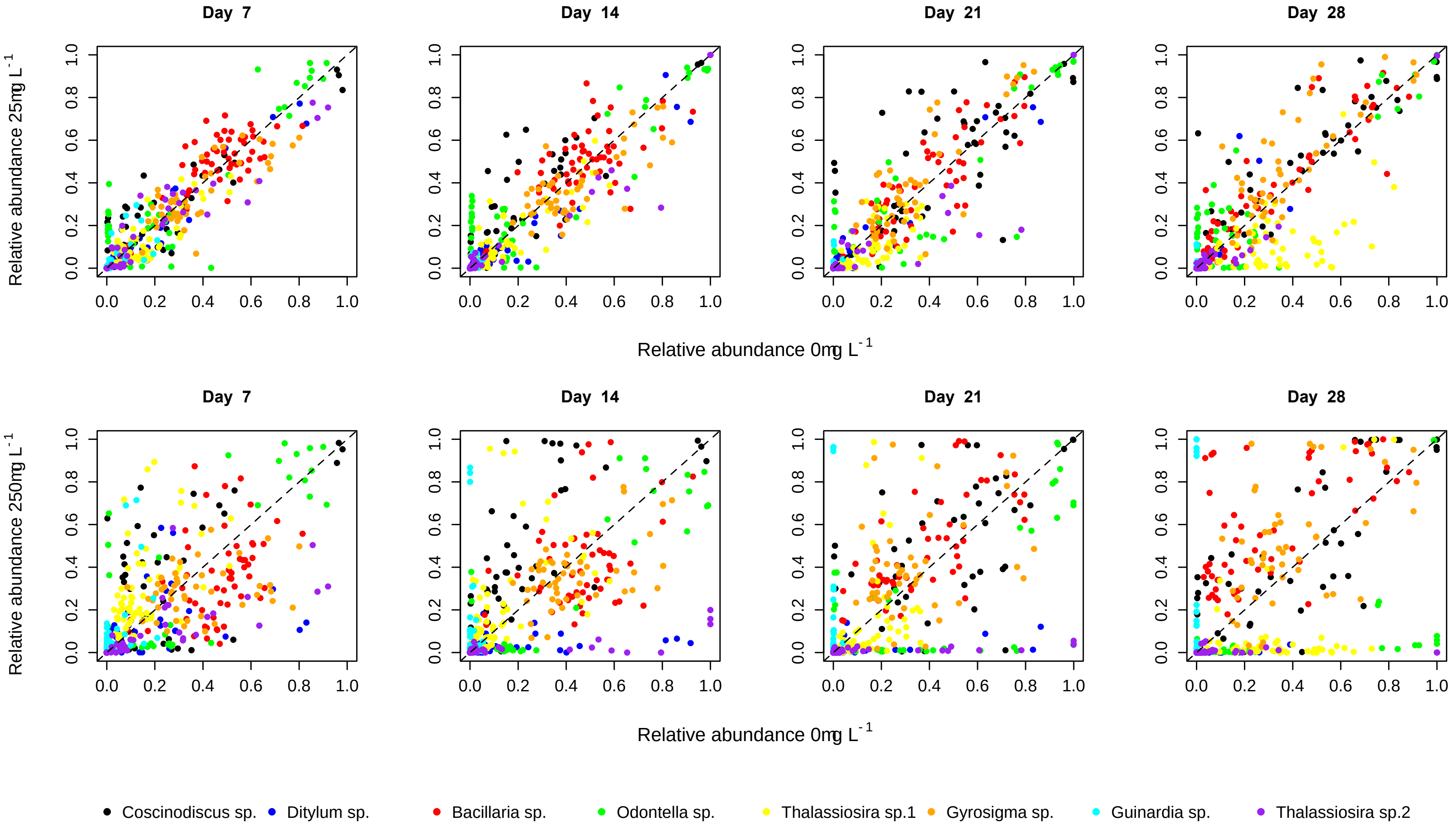

Supplementary Figure 1: Atrazine induced changes in relative species abundances. Changes in relative species abundances compared to the control treatment in the 25 μg L^{-1} (upper panels) and 250 μg L^{-1} (lower panels) atrazine treatment. Note that low stress (upper panels) induces small compositional differences with relative abundances close to the 1:1 line. High stress (lower panels), in contrast, induces larger compositional changes. Sensitive species decrease in abundance, lying under above the 1:1 line, whereas tolerant species increase and lie above this line.

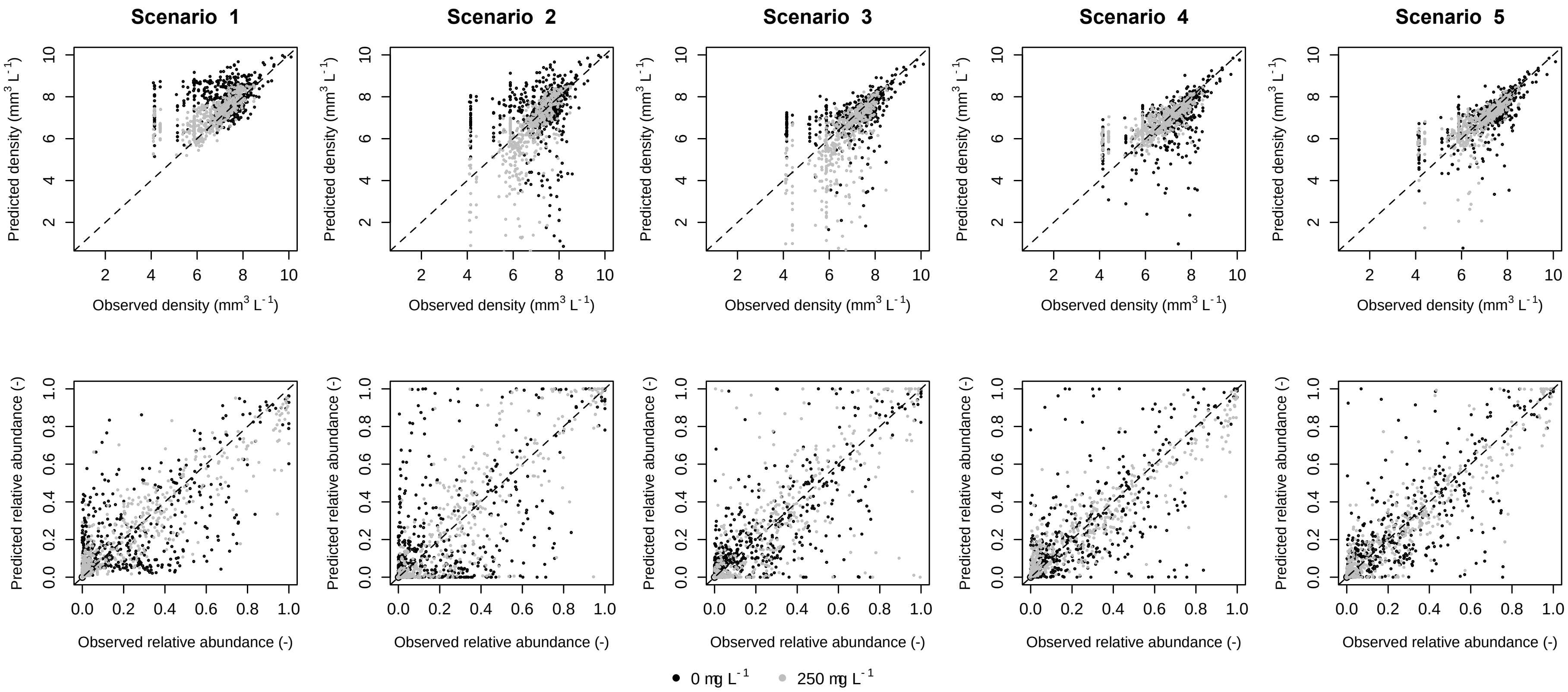

Supplementary Figure 2: Community model performance under different scenarios of stress-induced effects on the per-capita strength of species interactions. Predicted species densities and relative abundances plotted against the observed values from the biodiversity experiment for the 5 scenarios. Model predictions correspond better to observations when deviations from the dotted 1:1 line (i.e. perfect prediction) are smaller.

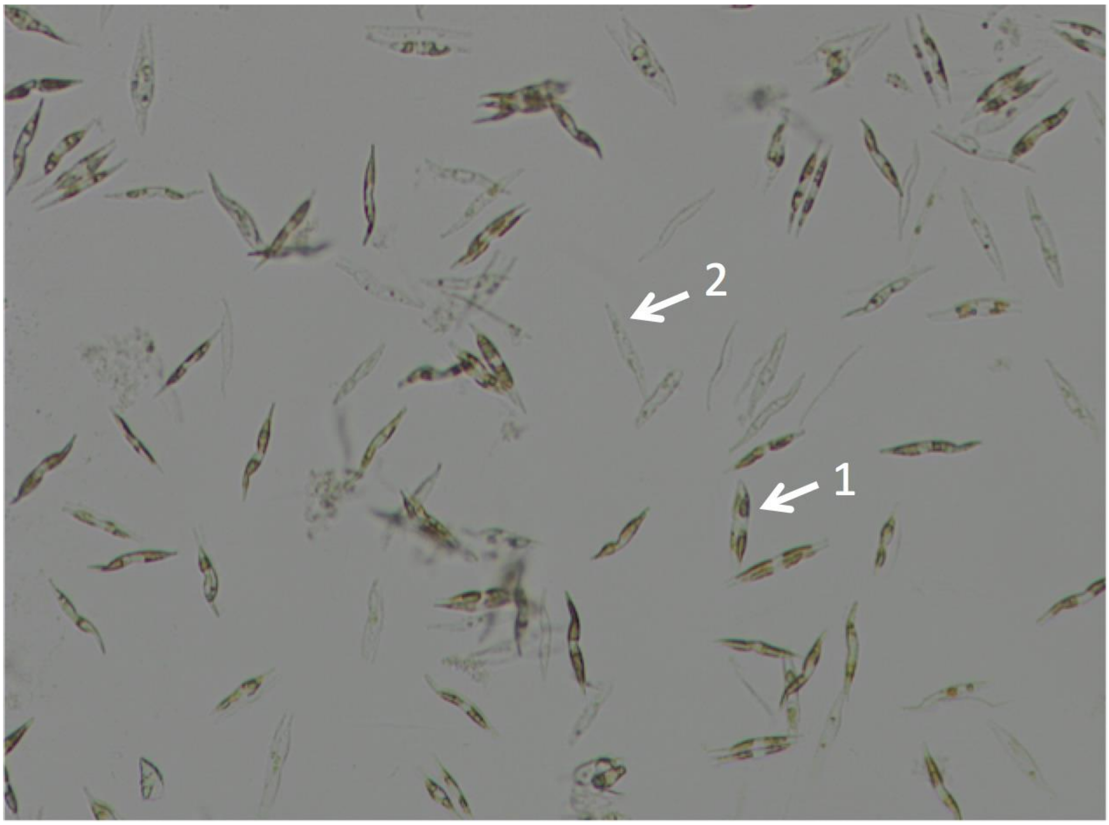

Supplementary Figure 3: Assessment of cell mortality Microscope image of a degrading diatom stock culture, illustrating the various stages ranging from living cells (1) to empty frustules (2). Note that this is heavily degraded stock culture to illustrate the different stages and does not represent any of the communities used in the experiment.

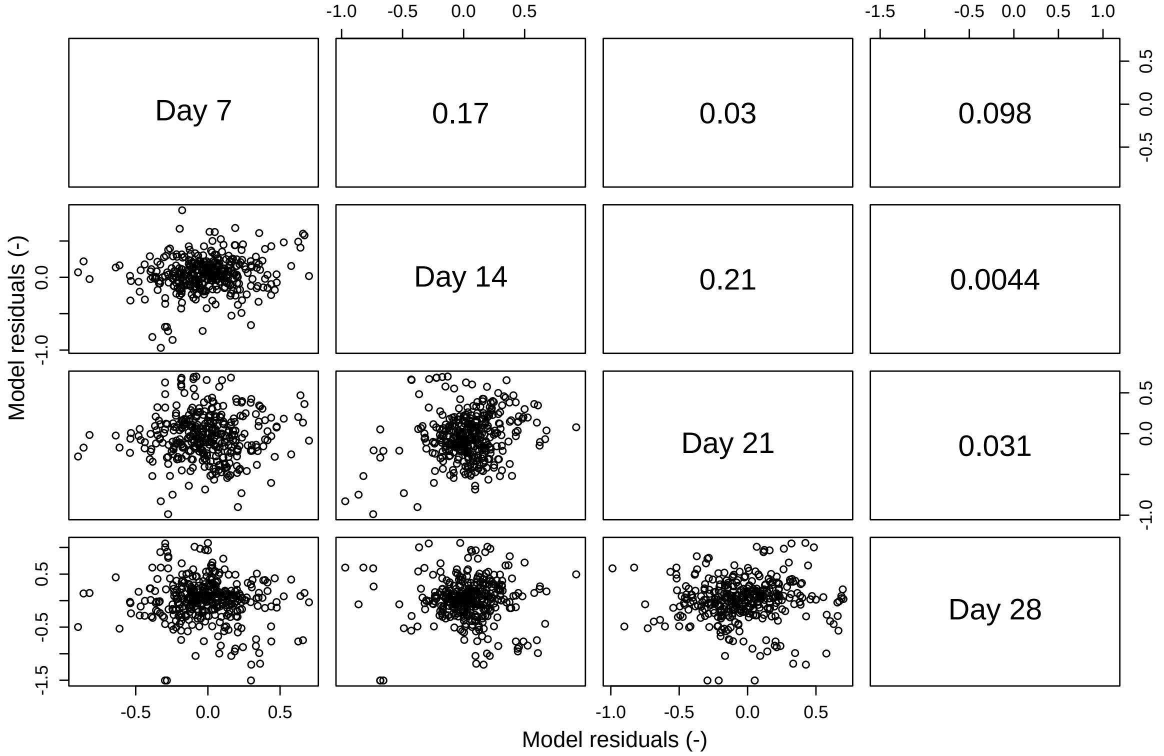

Supplementary Figure 4: Linear mixed effects model residual correlation for $\log_{10}$ biovolume. Pairs plot of the model residuals at each sampling day for the linear mixed effect model predicting the $\log_{10}$ biovolume as a function of the $\log_{10}$ diversity, atrazine concentration and time as fixed effects and species composition as a random effect (Table 1). Values in the upper triangle denote the correlation in model residuals between the different days.

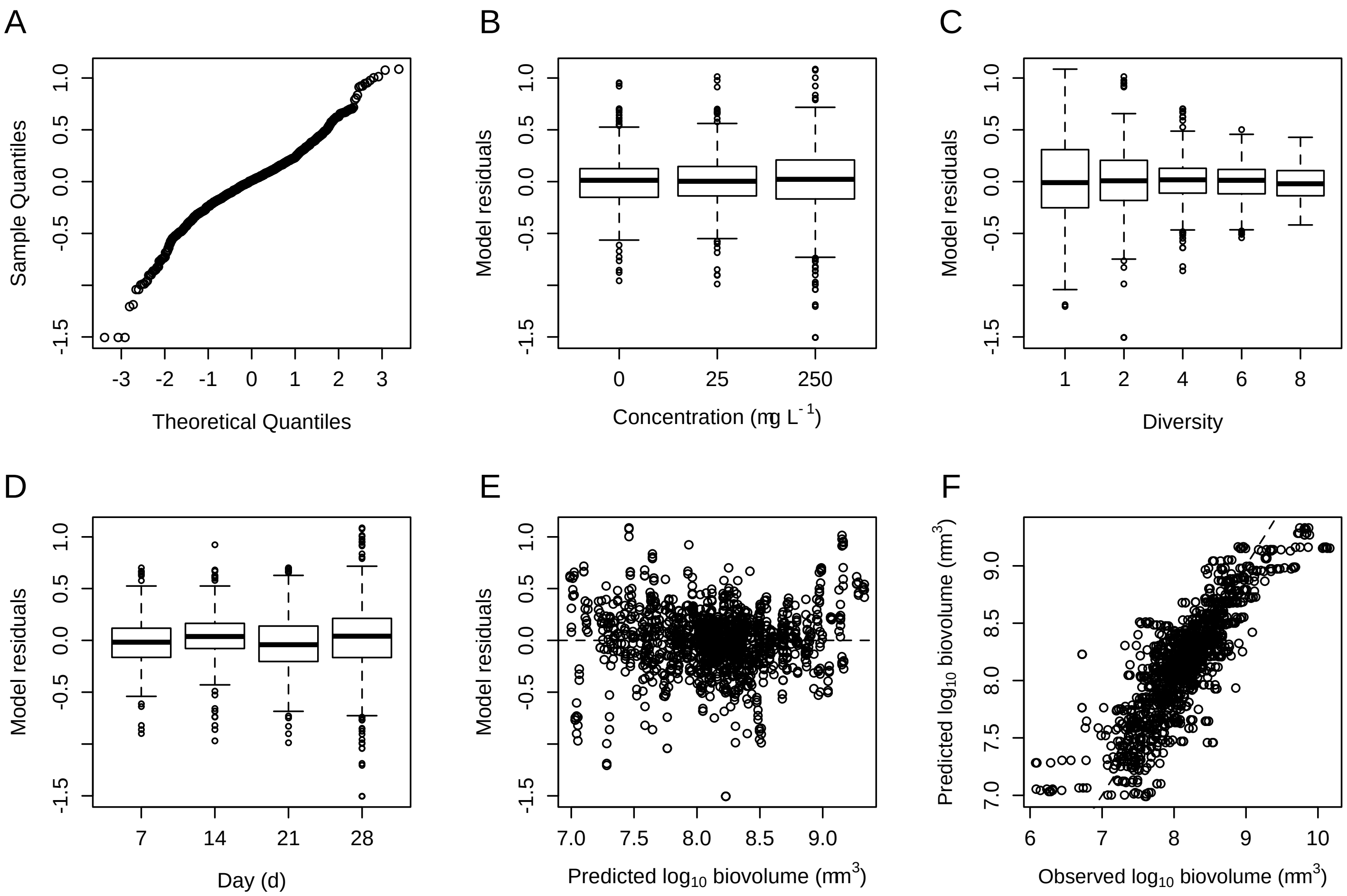

Supplementary Figure 5: Linear mixed effect model residual diagnostics for the $\log_{10}$ biovolume. Linear mixed effects model predicting the $\log_{10}$ biovolume as a function of $\log_{10}$ diversity, atrazine concentration and time as fixed effects and species composition as a random effect (Table 1).Model residuals are plotted as QQ-plot (A), against the fixed effects (atrazine concentration, B; diversity,C; and day,D) and plotted against the predicted $\log_{10}$ biovolume (E) to assess normality and homogeneity of model residuals. Model predictions are plotted against the observed log10 biovolumes to assess model performance.

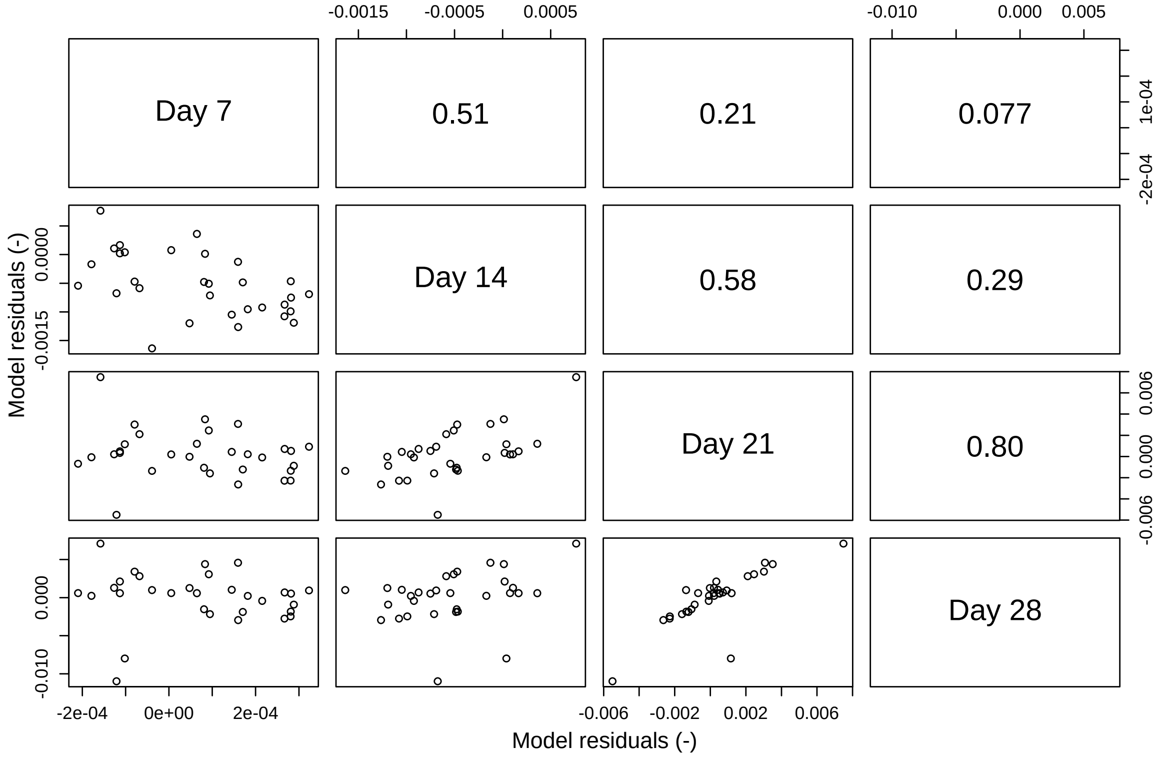

Supplementary Figure 6: Linear mixed effects model 1 residual correlation for changes in dominance effects Pairs plot of the model residuals at each sampling day for the linear mixed effect model predicting the changes in dominance effects as a function of the log_{10} diversity concentration and time as fixed effects and species composition as a random effect with temporal autocorrelation structure (Table 2, model1). Values in the upper triangle denote the correlation in model residuals between the different days.

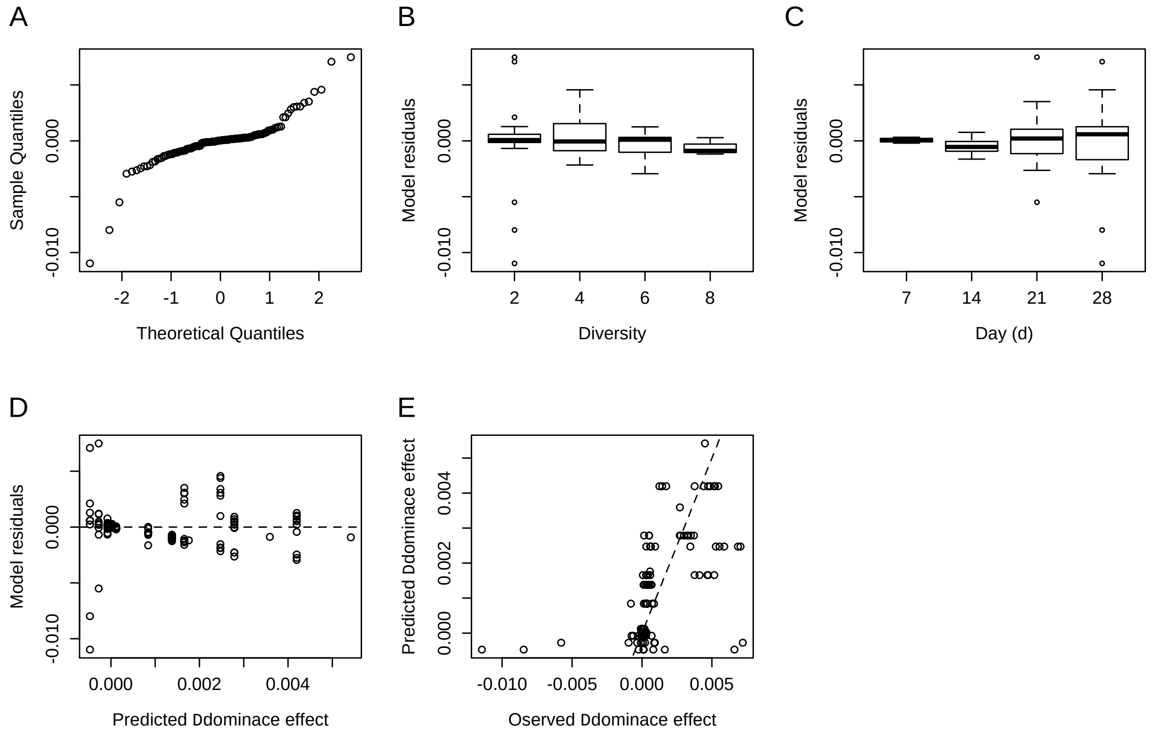

Supplementary Figure 7: Model 1 residual diagnostics for changes in dominance effects. Linear mixed effects model predicting atrazine-induced changes in the dominance effect (Δdominance effect) as a function of log10 diversity and time as fixed effects, community composition as random effects and a temporal autocorrelation structure (Table 2) Model residuals are plotted as QQ-plot (A), plotted against the fixed effects (Diversity, B and Day, C) and the predicted change in dominance effect (D) to assess normality and homogeneity of model residuals. Model predictions are plotted against the observed changes in dominance effects (E) to assess model performance.

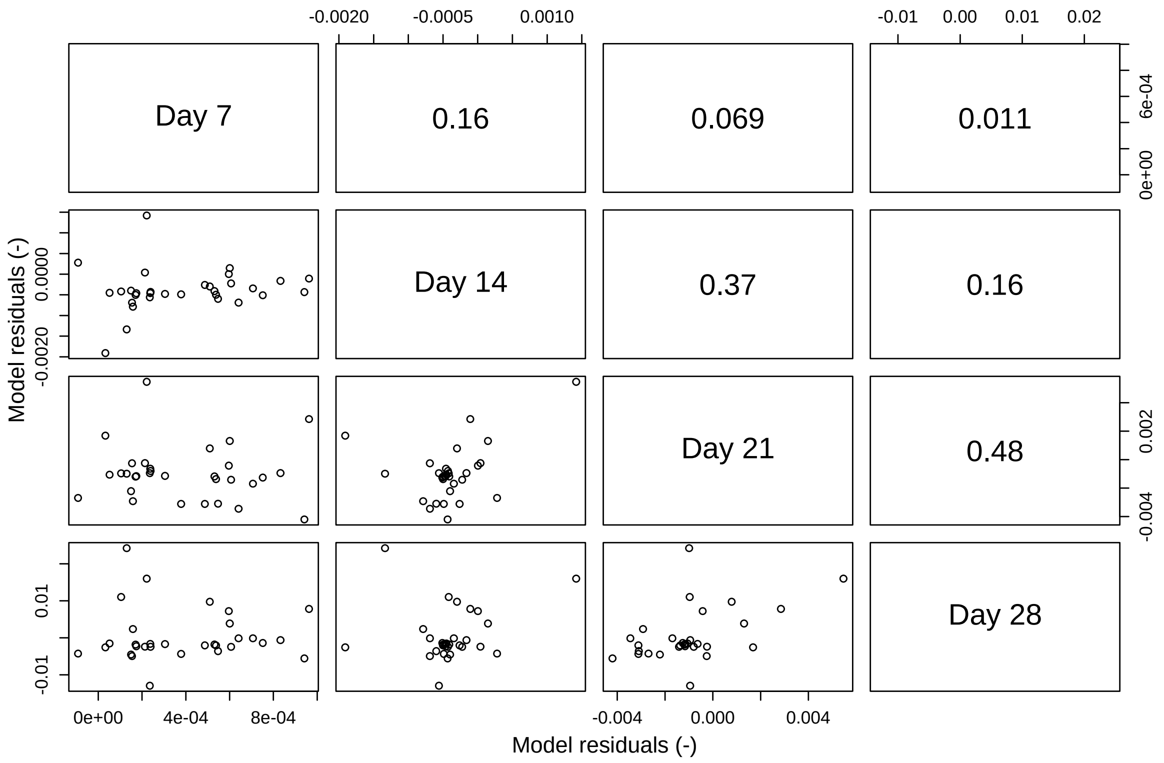

Supplementary Figure 8: Linear mixed effects model 1 residual correlation for changes in trait-dependent complementarity effects Pairs plot of the model residuals at each sampling day for the linear mixed effect model predicting the changes in trait-dependent complementarity effects (Δtrait.-dep. comp. effect) as a function of the $\log_{10}$ diversity concentration and time as fixed effects and species composition as a random effect with temporal autocorrelation structure (Table 2, model1). Values in the upper triangle denote the correlation in model residuals between the different days.

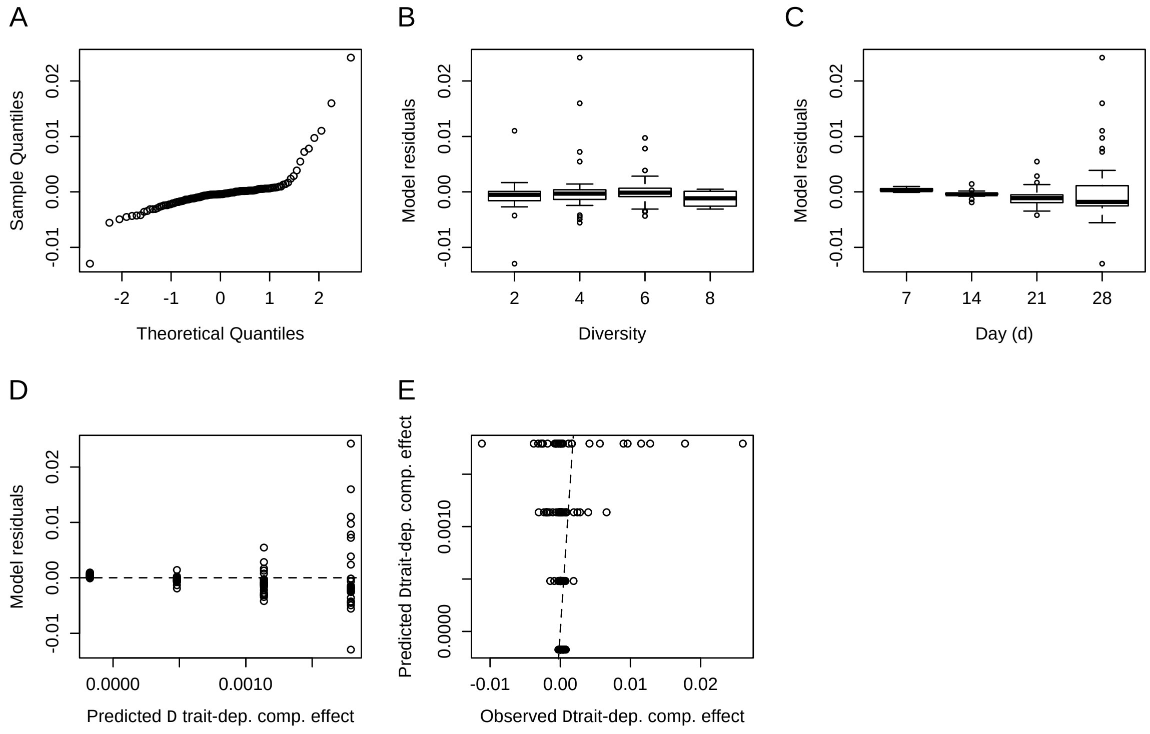

Supplementary Figure 9: Model 1 residual diagnostics for changes in trait-dependent complementarity effects. Linear mixed effects model predicting atrazine-induced changes in the dominance effect (Δtrait-dep. comp. effect) as a function of log10 diversity and time as fixed effects, community composition as random effects and a temporal autocorrelation structure (Table 2) Model residuals are plotted as QQ-plot (A), plotted against the fixed effects (Diversity, B and Day, C) and the predicted change in dominance effect (D) to assess normality and homogeneity of model residuals. Model predictions are plotted against the observed changes in trait-dependent complementarity effects (E) to assess model performance.

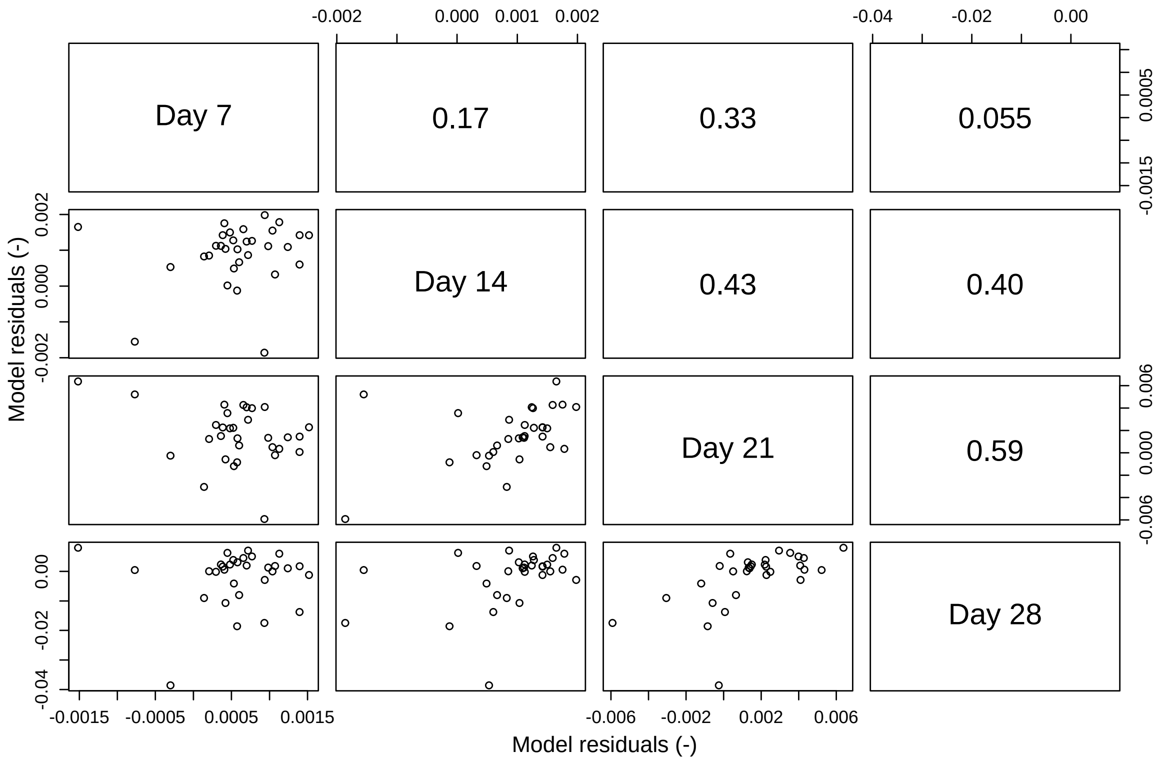

Supplementary Figure 10: Linear mixed effects model 1 residual correlation for changes in trait-independent complementarity effects Pairs plot of the model residuals at each sampling day for the linear mixed effect model predicting the changes in trait-dependent complementarity effects (Δtrait.-indep. comp. effect) as a function of the $\log_{10}$ diversity concentration and time as fixed effects and species composition as a random effect with temporal autocorrelation structure (Table 2, model1). Values in the upper triangle denote the correlation in model residuals between the different days.

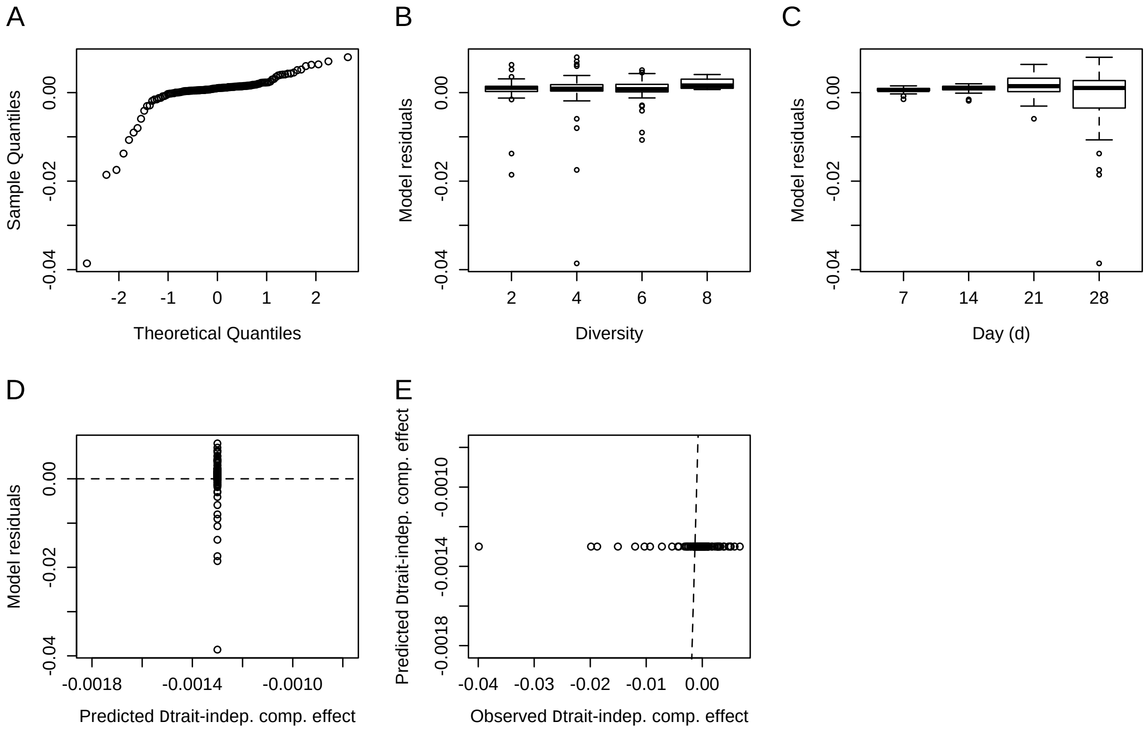

Supplementary Figure 11: Model 1 residual diagnostics for changes in trait-independent complementarity effects. Linear mixed effects model predicting atrazine-induced changes in the dominance effect (Δtrait-indep. comp. effect) as a function of log10 diversity and time as fixed effects, community composition as random effects and a temporal autocorrelation structure (Table 2) Model residuals are plotted as QQ-plot (A), plotted against the fixed effects (Diversity, B and Day, C) and the predicted change in dominance effect (D) to assess normality and homogeneity of model residuals. Model predictions are plotted against the observed changes in trait-independent complementarity effects (E) to assess model performance.

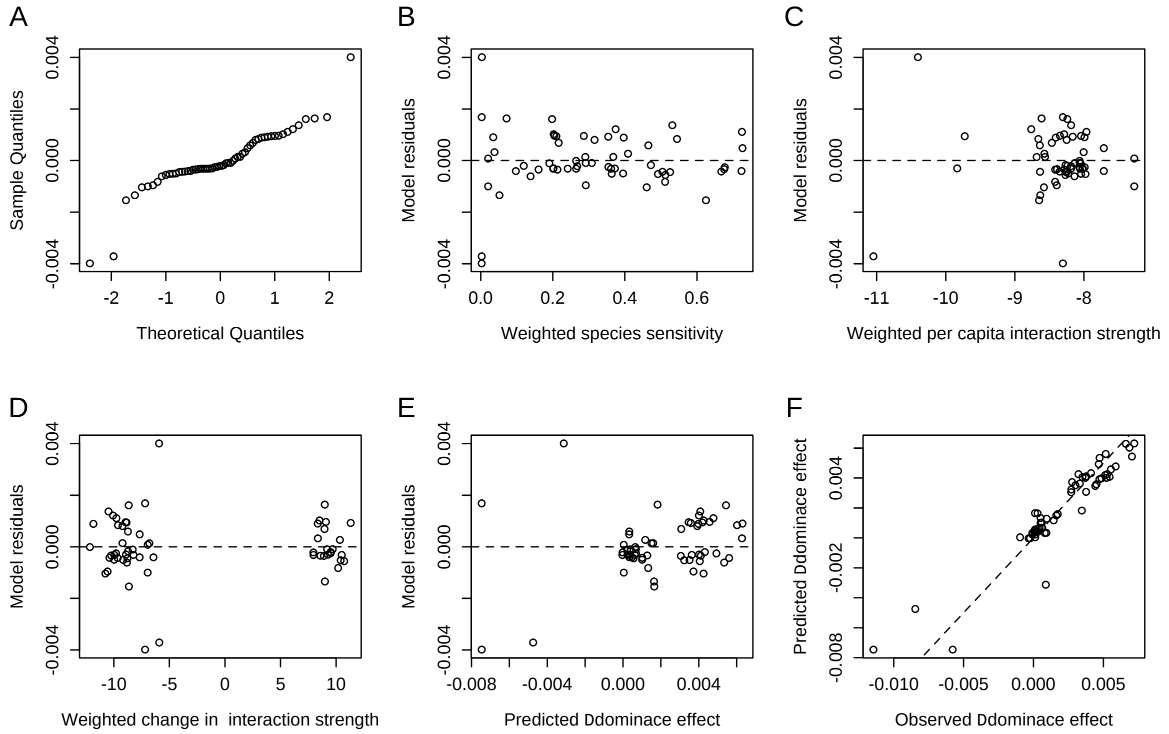

Supplementary Figure 12: Model 2 residual diagnostics for changes in dominance effects. Optimized linear mixed effects model predicting atrazine-induced changes in the dominance effect (Δdominance effect). Initial models included a function of the weighted mean species sensitivity to atrazine, per-capita interaction strengths and changes in per-capita interaction strengths as fixed effects and community composition as random effects (for optimal model structure, see Table 2) Model residuals are plotted as QQ-plot (A), plotted against the fixed effects (weighted mean species sensitivity, B; weighted mean per-capita interaction strength, C and weighted mean changes in the per-capita interaction strength, D) and the predicted change in dominance effect (E) to assess normality and homogeneity of model residuals. Model predictions are plotted against the observed changes in dominance effects (F) to assess model performance.

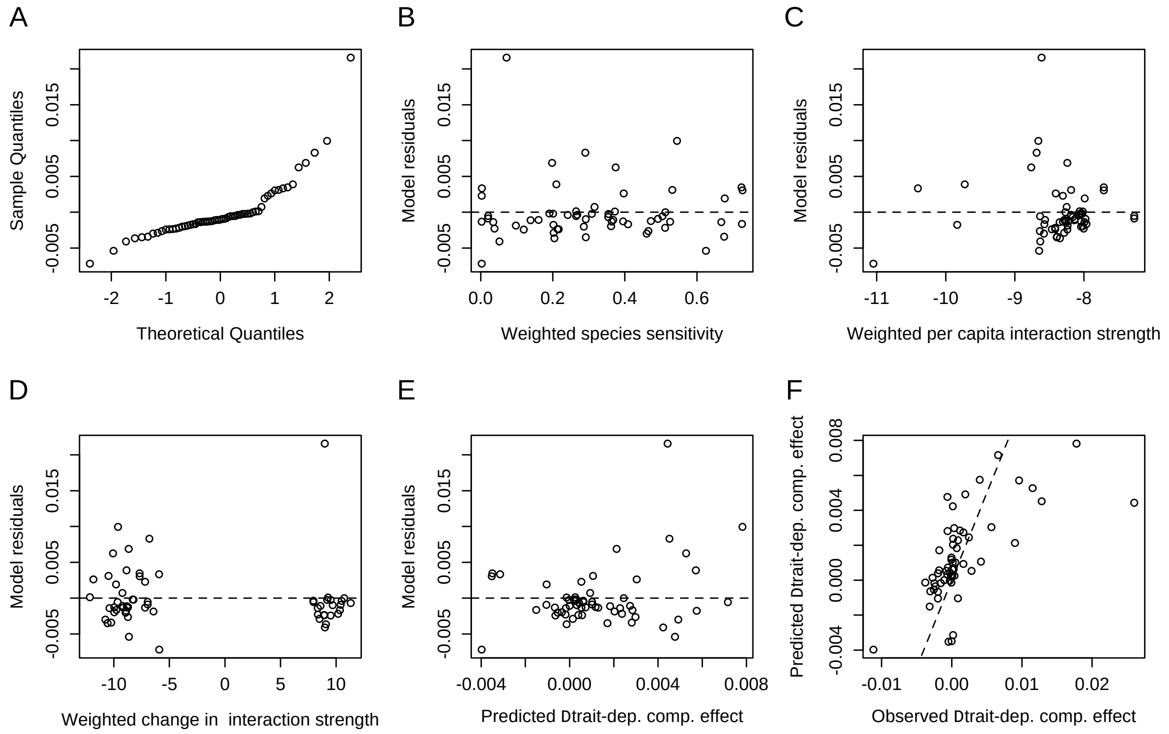

Supplementary Figure 13: Model 2 residual diagnostics for changes in trait-dependent complementarity effects. Optimized linear mixed effects model predicting atrazine-induced changes in the trait-dependent complementarity effect (Δtrait-dep. comp. effect). Initial models included a function of the weighted mean species sensitivity to atrazine, per-capita interaction strengths and changes in per-capita interaction strengths as fixed effects and community composition as random effects (for optimal model structure, see Table 2) Model residuals are plotted as QQ-plot (A), plotted against the fixed effects (weighted mean species sensitivity, B; weighted mean per-capita interaction strength, C and weighted mean changes in the per-capita interaction strength, D) and the predicted change in dominance effect (E) to assess normality and homogeneity of model residuals. Model predictions are plotted against the observed changes in trait-dependent complementarity effects (F) to assess model performance.

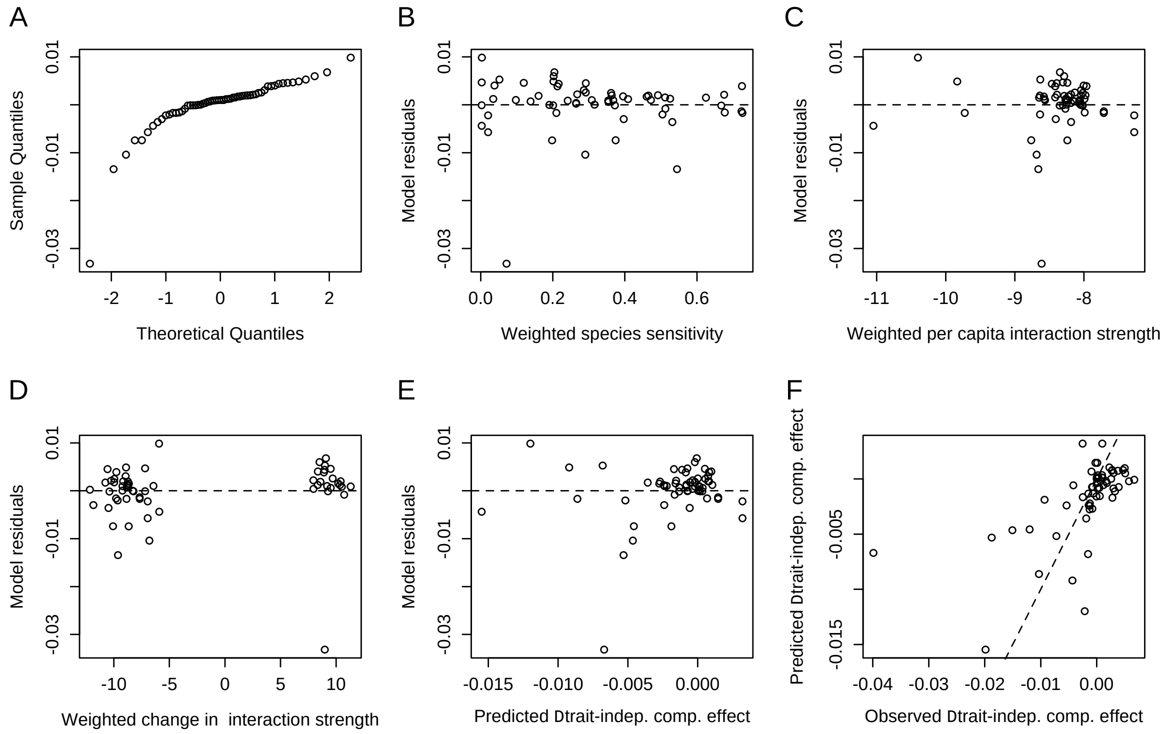

Supplementary Figure 14: Model 2 residual diagnostics for changes in trait-independent complementarity effects. Optimized linear mixed effects model predicting atrazine-induced changes in the trait-independent complementarity effect (Δtrait-dep. comp. effect). Initial models included a function of the weighted mean species sensitivity to atrazine, per-capita interaction strengths and changes in per-capita interaction strengths as fixed effects and community composition as random effects (for optimal model structure, see Table 2) Model residuals are plotted as QQ-plot (A), plotted against the fixed effects (weighted mean species sensitivity, B; weighted mean per-capita interaction strength, C and weighted mean changes in the per-capita interaction strength, D) and the predicted change in dominance effect (E) to assess normality and homogeneity of model residuals. Model predictions are plotted against the observed changes in trait-independent complementarity effects (F) to assess model performance.

Supplementary Tables

Supplementary Table 1 Estimates for the fixed effects and p-values for the linear mixed models including the weighted average sensitivity to atrazine stress (M_{250}/M_0), the average strength of per capita interactions (A_0) and the average change in per capita interaction strength (A_{250}-A_0) and all pairwise interactions as fixed effects. Community composition was included as a random effect.

			**Dominance Effect**	
	**DF**	**t-value**	**Estimate (standard error)**	**p-value**
Intercept	29	1.54	0.0216 (0.014)	0.14
M_{250}/M_0	24	-2.30	-0.0909 (0.039)	0.03
A_0	24	1.44	0.0024 (0.002)	0.16
A_{250}-A_0	24	0.25	0.0005 (0.002)	0.80
$M_{250}/M_0 \times A_0$	24	-2.31	-0.0111 (0.005)	0.03
$M_{250}/M_0 \times A_{250}$-$A_0$	24	-1.82	-0.0006 (0.0003)	0.08
$A_0 \times A_{250}$-A_0	24	0.15	0.00003 (0.00002)	0.89
			**Trait-dependent complementarity effect**	
	**DF**	**t-value**	**Estimate (standard error)**	**p-value**
Intercept	29	-0.12	-0.0024 (0.02)	0.91
M_{250}/M_0	24	-3.07	-0.1723 (0.06)	0.005
A_0	24	-0.15	-0.0004 (0.002)	0.88
A_{250}-A_0	24	-0.84	-0.0024 (0.003)	0.41
$M_{250}/M_0 \times A_0$	24	-3.08	-0.0210 (0.007)	0.005
$M_{250}/M_0 \times A_{250}$-$A_0$	24	-0.62	-0.0003 (0.0005)	0.54
$A_0 \times A_{250}$-A_0	24	-0.86	-0.0003 (0.0003)	0.40
			**Trait-independent complementarity effect**	
	**DF**	**t-value**	**Estimate (standard error)**	**p-value**
Intercept	29	2.63	0.069 (0.03)	0.01
M_{250}/M_0	24	1.09	0.076 (0.07)	0.29
A_0	24	2.71	0.009 (0.003)	0.01
A_{250}-A_0	24	1.51	0.005 (0.007)	0.14
$M_{250}/M_0 \times A_0$	24	1.07	0.009 (0.009)	0.30
$M_{250}/M_0 \times A_{250}$-$A_0$	24	0.58	0.0004 (0.0006)	0.57
$A_0 \times A_{250}$-A_0	24	1.53	0.0007 (0.0004)	0.14

Supplementary Table 2: Estimated population parameters for the 8 species used in the biodiversity experiment. V is the cell volume, K_0 and K_{250} are the carrying capacities, μ_0 and μ_{250} are the intrinsic growth rates at 0 and 250 µg L-1 Atrazine, respectively

Code	Species	V (µm^3)	μ_0 (d^{-1})	μ_{250} (d^{-1})	K_0 (µm^3)	K_{250} (µm^3)
1	*Coscinodiscus sp.*	367008	0.33	0.22	948 839 324	314883704
2	*Ditylum sp.*	24757	0.27	0.01	862 505 671	4951400
3	*Bacillaria sp.*	6448	0.51	0.20	406 957 000	348711139
4	*Odontella sp.*	752767	0.24	0.01	10 583 784 289	55990944
5	*Thalassiosira sp.1*	25727	0.28	0.15	108 096 248	43707354
6	*Gyrosigma sp.*	3940	0.19	0.10	651 492 127	66671926
7	*Guinardia sp.*	14001	0.19	0.19	1 176 672 117	28473370
8	*Thalassiosira sp.2*	12556	0.43	0.01	629 079 021	15554781

Supplementary Table 3: Community assemblages for diversity levels 2, 4 and 6. Numbers refer to the species code in supplementary table 1. Assemblages at diversity 1 and 8 are not given here since all possible combinations were used for these levels

Diversity = 2	Diversity = 4	Diversity = 6
1+3	1+2+4+8	1+2+3+4+5+7
2+4	2+4+5+6	1+2+4+5+6+7
7+8	3+4+5+8	1+3+4+5+6+7
1+7	2+3+4+5	1+2+3+5+6+7
3+5	1+2+3+6	1+2+3+5+6+8
5+6	3+5+7+8	2+3+5+6+7+8
4+6	2+3+4+7	1+3+4+5+6+8
3+8	1+5+6+8	2+3+4+5+6+7
2+5	1+3+5+6	2+4+5+6+7+8
1+3	1+2+4+7	3+4+5+6+7+8

Supplementary Table 4: Metadata for the file *data.txt* containing the experimental data

Column caption	Value	Unit
composition	Species composition of the community	-
day	Day of the measurement	-
replicate	Technical replicate	-
concentration	Atrazine treatment	µg L-1
1	Density species 1	cells mL-1
2	Density species 2	cells mL-1
3	Density species 3	cells mL-1
4	Density species 4	cells mL-1
5	Density species 5	cells mL-1
6	Density species 6	cells mL-1
7	Density species 7	cells mL-1
8	Density species 8	cells mL-1
NO_3^-	Nitrate concentration	mg L-1
PO_4^{-2}	Phosphate concentration	mg L-1
Si	Silica concentration	mg L-1
Atrazine	Atrazine concentration	µg L-1
